# Y-chromosome haplogroup architecture confers susceptibility to azoospermia factor c microrearrangements: a retrospective study

**DOI:** 10.3325/cmj.2019.60.273

**Published:** 2019-06

**Authors:** Maja Kuzmanovska, Predrag Noveski, Marija Terzic, Toso Plaseski, Katerina Kubelka-Sabit, Vanja Filipovski, Slobodan Lazarevski, Emilija Sukarova Stefanovska, Dijana Plaseska-Karanfilska

**Affiliations:** 1 Research Center for Genetic Engineering and Biotechnology “Georgi D. Efremov,” Macedonian Academy of Science and Arts, Skopje, North Macedonia; 2 Clinical Hospital “Acibadem Sistina,” Skopje, North Macedonia; 3 Clinic of Endocrinology and Metabolic Disorders, Faculty of Medicine, Skopje, North Macedonia; Kuzmanovska et al: Y haplogroups and azoospermia factor c microrearrangements

## Abstract

**Aim:**

To assess the association between azoospermia factor c microrearrangements and semen quality, and between Y-chromosome background with distinct azoospermia factor c microrearrangements and semen quality impairment.

**Methods:**

This retrospective study, carried out in the Research Center for Genetic Engineering and Biotechnology “Georgi D. Efremov,” involved 486 men from different ethnic backgrounds referred for couple infertility from 2002-2017: 338 were azoospermic/oligozoospermic and 148 were normozoospermic. The azoospermia factor c microrearrangements were analyzed with sequence tagged site and sequence family variant markers, quantitative fluorescent polymerase chain reaction, and multiplex ligation probe amplification analysis. The Y-haplogroups of all participants were determined with direct single nucleotide polymorphism typing and indirect prediction with short tandem repeat markers.

**Results:**

Our participants had two types of microdeletions: gr/gr and b2/b3; three microduplications: b2/b4, gr/gr, and b2/b3; and one complex rearrangement gr/gr deletion + b2/b4 duplication. Impaired semen quality was not associated with microrearrangements, but b2/b4 and gr/gr duplications were significantly associated with haplogroup R1a (*P* < 0.001 and *P* = 0.003, respectively) and b2/b3 deletions with haplogroup E (*P* = 0.005). There were significantly more b2/b4 duplication carriers in Albanians than in Macedonians with haplogroup R1a (*P* = 0.031).

**Conclusion:**

Even though azoospermia factor c partial deletions/duplications and Y-haplogroups were not associated with impaired semen quality, specific deletions/duplications were significantly associated with distinct haplogroups, implying that the Y chromosome background may confer susceptibility to azoospermia factor c microrearrangements.

There is intensive research into the importance of the Y chromosome in sex determination and spermatogenesis. The Y chromosome has a unique structure, with about 30% of the male-specific region (MSY) composed of massive, near-perfect amplicons. The MSY is remarkable for its structural complexity and the propensity to undergo massive rearrangements, especially in the azoospermia factor c (AZFc) region (1). The palindromic and repetitive nature of the AZFc region makes it highly susceptible to genomic rearrangements mediated by non-allelic homologous recombination, leading to deletions, duplications, and copy number variations (2-7). The absence of the entire AZFc region is a major cause of azoospermia or oligozoospermia (8-10). However, it is unclear to what extent semen quality is impaired by partial deletions in this region (ie, gr/gr and b2/b3 deletions) (11,12). For instance, the most common partial deletion (8), the gr/gr deletion, removes almost half the gene content of the AZFc region, involving two copies of the *Deleted in Azoospermia* (*DAZ*) gene and one copy of *Chromodomain protein on Y, 1* (*CDY1*) (13). Individuals with gr/gr deletions have a lower average sperm count than those without these deletions (14-18). The b2/b3 deletion removes the 1.8 Mb region, including two *DAZ* copies and one *CDY1* copy. High frequencies of this deletion were first reported in individuals with haplogroup N in northern Eurasian populations, indicating a founder mutation (5,19).

The gr/gr deletions present a risk factor for impaired semen quality in some populations and Y haplogroups, but not in others (4,16,20). For instance, haplogroups Q1 and D2b, common in China and Japan, have uniformly deleted gr/gr region, without decrease in semen quality (20). This indicates that the Y chromosome background may modulate the penetrance of this partial deletion (20,21). The b2/b3 deletion is strongly associated with impaired semen quality in the Han Chinese population and Dravidians, and many deletion carriers were found in haplogroups other than N (22-24). Thus, b2/b3 deletion may confer infertility risk when occurring outside this haplogroup (5,25). Another important question is whether increased gene dosage of the AZFc region may affect fertility, since an “optimal” copy number is required for normal spermatogenesis. Furthermore, a number of research groups attempted to find the connection between the variable phenotypic effects of the AZFc deletions and a distinctive set of genes, however such association still remains dubious (20,23,26). No data has been provided for the potential damaging effect of b2/b4 duplication on semen quality, even though the b2/b4 deletion is a well-known cause for male infertility (6).

We hypothesize that the AZFc microrearrangements confer the risk for impaired semen quality, and that the Y chromosome background influences the occurrence of specific rearrangements among men referred for couple infertility. Accordingly, our aim was to determine the frequency of AZFc partial deletions and duplications and their association with impaired semen quality in Macedonian and Albanian population and to evaluate the association of Y-chromosome background with distinct AZFc microrearrangements and semen quality impairment. Additionally, we identified whether deletions of specific *DAZ* and *CDY1* gene copies determined the phenotypic manifestations of AZFc deletions.

## PARTICIPANTS AND METHODS

### Participants

The study involved 338 men with impaired semen quality referred to our laboratory on the basis of semen analysis. The patients were divided into two groups according to their sperm count: azoospermic group, consisting of 154 patients (no sperm in the ejaculate even after centrifugation), and oligozoospermic group, consisting of 184 patients (sperm concentration 0.1-20 × 10^6^/mL). The classification was based on the WHO Laboratory Manual for Examination and Processing of Human Semen from 1999 (27), in order to make our results comparable to the results of the published meta-analyses (15,18,25). Additionally, the number of oligozoospermic patients with sperm concentration from 15-20 × 10^6^/mL was too low to be included into statistical analysis. The control group consisted of 148 normozoospermic men, who were recruited from infertile couples in which the female factor was responsible for the couples’ inability to conceive. The fertility status of controls was unknown, since we assessed the effect of AZFc rearrangements on semen quality, not the men’s ability to conceive a child. Our study is retrospective, since all of the examined participants were directed to our research center for genetic investigation from three different clinics in the period 2002-2017. The analyses were carried out in the Research Centre for Genetic Engineering and Biotechnology “Georgi D. Efremov.” The exclusion criteria were karyotype abnormalities, complete AZF deletions, or obstructive azoospermia. A written informed consent was obtained from each participant for the molecular investigations and data publication. The study protocol was approved by the Ethics Committee of the Macedonian Academy of Science and Arts (No. 09-1047/5, May 20, 2016).

The study population was of different ethnic origin, which is why we divided the participants in three groups: Macedonians, Albanians, and others (small number of patients including Serbs, Croats, Romani, and Turks). The number of examined patients in each ethnic group reflects the demographic composition of our country, and the frequency of normozoospermic men in every ethnic group reflects the percentage of azoo/oligoozoospermic patients (Table 1).

**Table 1:** Ethnic composition of study participants

Nationality	Spermatogenic status, n (%)		
	azoospermia (n = 154)	oligozoospermia (n = 184)	normozoospermia (n = 148)
Macedonian	95 (61.7)	129 (70.1)	103 (69.6)
Albanian	40 (26.0)	36 (19.6)	33 (22.3)
Others*	19 (12.3)	19 (10.3)	12 (8.1)

*Others comprise Serbs, Croats, Romani, and Turks.

### AZFc sequence tagged site and sequence family variant analysis

Basic genetic analysis was performed using the conventional Y chromosome sequence-tagged site (STS) markers, sY1291, sY1191, and sY1192, as previously described (4). The absence of sY1291 represents a gr/gr deletion and the absence of sY1191 and sY1192 represents a b2/b3 deletion. Beside the STS markers used in our study, additional markers are available for the detection of gr/gr and b2/b3 deletions (10).

The two *DAZ* (*DAZ 1/2* and *DAZ 3/4*) and *CDY* (*CDY1a* and *CDY1b*) gene clusters were differentiated using sequence family variant (SFV) analysis (28,29). Briefly, we amplified the SFV at the sequence-tagged sites, sY581, sY586, and sY587, to analyze the *DAZ* gene copy deletions. The amplified products were digested overnight using *Sau*3AI*, TaqI,* and *DraI* restriction enzymes, respectively (28). The sY581 SFV discriminates *DAZ 1/4* from *DAZ 2/3*, the sY586 discriminates *DAZ 2* from *DAZ 1/3/4*, whereas sY587 discriminates *DAZ 1/2* from *DAZ 3/4* (28-30). CDY1-7750 was amplified, and the PCR products were digested with the restriction enzyme, PvuII, differentiating between *CDY1a* and *CDY1b* (29). The digested fragments were separated by 2.0% agarose gel electrophoresis in order to characterize the six major deletion types.

### Gene dosage analysis

Our laboratory had developed a multiplex quantitative fluorescence polymerase chain reaction (QF-PCR) method for simultaneous detection of sex chromosomal aneuploidies, AZF deletions, and partial AZFc deletions/duplications (31,32). The results obtained from the 11-plex QF-PCR were primarily used to eliminate patients with chromosomal abnormalities and complete AZF deletions. Furthermore, for the patients with partial AZFc deletions/duplications we calculated the relative ratio of peaks to determine the copy number of *DAZ* and *CDY*. For quantification of the *DAZ* region, a fragment of intron 10 of the *DAZ* gene (208 bp) was co-amplified with the homologous autosomal locus *DAZL* on chromosome 3 (211 bp or 251 bp). The ratio of the two *CDY1* genes in the AZFc region and the two *CDY2* genes in the AZFb region was also determined as previously described (31,32).

### Multiplex ligation-dependent probe amplification analysis

Multiplex ligation-dependent probe amplification (MLPA) analysis was performed using the MLPA P360-A1 Y-Chromosome Microdeletions kit (MRC-Holland, Amsterdam, The Netherlands). The probes detect copy number variations in the AZFa, AZFb, and AZFc regions. MLPA analysis included only the participants with previously detected deletion or duplication. It was performed according the manufacturer’s instructions on both patients and controls. The samples were separated on the ABI 3500 Genetic Analyzer, and the chromatogram analysis was performed with Coffalyser.NET (MRC Holland, *https://support.mlpa.com/kb/coffalyser-net*). Sequences showing aberrant copy numbers were identified by comparing the peak area of our sample with the peak area of the reference samples. A decrease of the signal of the probes targeting two sequences by 50% compared with the referent signal indicates that one of the probes was deleted. Likewise, the decrease of the signal of the probes targeting 3 sequences by 33% or 67% indicates that one or two of the aimed probes was deleted, respectively.

### Y chromosome haplogrouping

The Y chromosome haplogrouping was performed with two different methodologies: a) direct Y chromosome single nucleotide polymorpishm (SNP) typing with SNaPShot minisequencing (33) and b) indirect prediction with Y short tandem repeat (STR) markers typing carried out with AmpFlSTR® Yfiler® PCR Amplification Kit (Thermo Fisher Scientific, Waltham, MS, USA). The predictions were made with two web-based software packages: 1) Whit Athey’s Haplogroup Predictor (34), which predicts the haplogroup by comparing the analyzed Y STR markers with those of a larger subject group with previously determined Y chromosome haplogroups (35,36) 2) Jim Cullen’s Haplogroup Predictor (37). The haplogroups were directly determined for 194 participants (62 azoospermic, 81 oligozoospermic, and 51 normozoospermic) and indirectly for all 486 participants. We observed a total agreement of the results obtained with these methods, thus giving us reasonable confidence in the results yielded with the Y STR-based methodology for the remaining 292 participants. Additional evidence for the accuracy of the used prediction software packages is provided by another recently published study (38). The haplogroups were named according to the 2008 Y-DNA haplogroup tree by the International Society of Genetic Genealogy (ISOGG). In addition, we present the haplogroup names along with their corresponding markers, facilitating the conversion of the haplogroup names to other versions of the ISOGG tree. Therefore, for the readability purpose, the markers were omitted from the haplogroup names in the rest of the manuscript.

### Statistical analysis

Pearson χ^2^ was performed with the R statistical software. Fischer exact test with Benjamin & Hochberg (BH) correction for multiple testing was carried out with “fifer” package (39), while data manipulation and visualization were performed with the “tidyverse” (40) collection of packages in R (41). Power analysis was conducted *post- hoc* using the “pwr” package with the alpha level of 0.05 (42), while effect sizes were calculated as Cramer’s Phi (square root of χ^2^/n, where n is number of the participants) (43). The analyses for the association of the microrearrangements with impaired semen quality were underpowered, while analyses for the association of different rearrangements with the five major haplogroups showed adequate statistical power of over 80% when assuming large effect size. The level of significance was set at *P* ≤ 0.05.

## RESULTS

### Prevalence of the AZFc deletions and duplications in different ethnic groups

Our participants had six different types of AZFc rearrangements: b2/b4 duplication, gr/gr deletion, gr/gr duplication, b2/b3 deletion, b2/b3 duplication, and gr/gr deletion followed by b2/b4 duplication. There were no significant differences in the frequencies of the detected rearrangements between azoospermic/oligozoospermic patients and controls (Table 2).

**Table 2:** Distribution of deletions and duplications among the examined patients and controls

	Spermatogenic status			*P* (Fisher exact test)		
Type of rearrangement, n (%)	azoospermia (n = 154)	oligozoospermia (n = 184)	normozoospermia (n = 148)	azoospermia vs normozoospermia	oligozoospermia vs normozoospermia	azoospermia vs oligozoospermia
**b2/b3 deletion**	2 **(**1.3**)**	3 **(**1.6**)**	2 **(**1.3**)**	>0.999	>0.999	>0.999
**b2/b3 duplication**	4 **(**2.6**)**	4 **(**2.2**)**	1 **(**0.7**)**	0.371	0.386	>0.999
**b2/b4 duplication**	7 **(**4.5**)**	11 **(**6.0**)**	9 **(**6.1**)**	0.613	>0.999	0.632
**gr/gr deletion + b2/b4 duplication**	0 (0)	0 **(0)**	1 **(**0.7**)**	-	-	-
**gr/gr deletion**	5 **(**3.2**)**	4 **(**2.2**)**	4 **(**2.7**)**	>0.999	>0.999	0.736
**gr/gr duplication**	0 **(0)**	5 **(**2.7**)**	4 **(**2.7**)**	0.056	>0.999	0.065

The most commonly observed microrearrangement was the b2/b4 duplication, which was detected almost twice as frequently among Albanians (9/109, 8.3%) than Macedonians (18/327, 5.5%), but was not found in other ethnicities (Table 3). The gr/gr duplication was detected in 8 Macedonian participants, with a similar frequency in oligozoospermic and normozoospermic men, and in one oligozoospermic man of Serbian origin. This partial duplication was not detected among Albanians and in the azoospermic subgroup. The b2/b3 deletion was detected in all three ethnic groups, while the b2/b3 duplication was detected only in Macedonian and Albanian participants. Both of the b2/b3 rearrangements were detected in all subgroups with different degrees of semen impairment.

**Table 3:** Frequency and distribution of the azoospermia factor c partial deletions and duplications among participants of different ethnic origin with different semen quality

	Macedonians (n = 327)			Albanians (n = 109)			Others* (n = 50)			Total (n = 486)
**Type of rearrangement, n (%)**	**azoospermia** **(n = 95)**	**oligozoospermia (n = 129)**	**normozoospermia (n = 103)**	**azoospermia (n = 40)**	**oligozoospermia (n = 36)**	**normozoospermia (n = 33)**	**azoospermia (n = 19)**	**oligozoospermia (n = 19)**	**normozoospermia (n = 12)**
**b2/b4 duplication**	5 (5.3)	9 (7.0)	4 (3.9)	2 (5.0)	2 (5.5)	5 (15.1)	-	-	-	27 (5.5)
**gr/gr deletion**	4 (4.2)	4 (3.1)	4 (3.9)	1 (2.5)	-	-	-	-	-	13 (2.7)
**gr/gr duplication**	-	4 (3.1)	4 (3.9)	-	-	-	-	1 (5.3)	-	9 (1.8)
**b2/b3 deletion**	1 (1.0)	-	1 (1.0)	-	2 (5.5)	1 (3.0)	1 (5.3)	1 (5.3)	-	7 (1.4)
**b2/b3 duplication**	3 (3.2)	2 (1.5)	1 (1.0)	1 (2.5)	2 (5.5)	-	-	-	-	9 (1.8)
**gr/gr del.+ b2/b4 dupl.**	-	-	1 (1.0)	-	-	-	-	-	-	1 (0.2)
**Normal**	82 (86.3)	110 (85.3)	88 (85.4)	36 (90.0)	30 (83.3)	27 (81.8)	18 (94.7)	17 (89.5)	12 (100)	420 (86.4)

*Others comprise Serbs, Croats, Romani, and Turks.

### Distribution of AZFc deletions and duplications among different Y-haplogroups

Our participants belonged to 17 different Y haplogroups, but more than 91% of them belonged to 5 major haplogroups (I, E, J, R1a, and R1b). No difference in the haplogroup distribution was observed between the normozoospermic men included in this study and the general population of North Macedonia (33) (*P* = 0.235, χ^2^ test), suggesting that the control group is representative of the population of the whole country. Partial deletions and duplications were detected in 4 of the 5 major haplogroups (Supplementary Figure 1(supplementary Figure 1)) and in 10 different haplogroups (Supplementary Figure 2(supplementary Figure 2)).

The b2/b4 duplications and gr/gr deletions were observed in seven different haplogroups, and b2/b3 deletions in only two. Interestingly, the gr/gr deletions were detected almost exclusively among Macedonians, while the b2/b4 duplications were detected in both Macedonians and Albanians. The b2/b4 duplication was detected in both these ethnicities in haplogroup R1a, but not in other six haplogroups (Supplementary Figure 2(supplementary Figure 2)).

In haplogroups E and J, four different types of rearrangements were detected (both b2/b3 and gr/gr rearrangements), while in haplogroups I and R1a only gr/gr deletions/duplications were detected (Supplementary Figure 1(supplementary Figure 1) and Supplementary Figure 2(supplementary Figure 2)). There was a striking difference in the occurrence rate of AZFc rearrangements between two phylogenetically close haplogroups, R1a and R1b. Namely, almost half of the participants (45.2%) from the haplogroup R1a carried AZFc rearrangements, compared with none of the participants from the R1b haplogroup. The same was observed for haplogroups J2 and J1, although this finding should be taken with caution due to the small number of participants from haplogroup J1 (Table 4).

**Table 4:** Distribution and frequency of partial deletions and duplications within haplogroups

Haplogroups-markers, n (%)	Type of rearrangement						Normal	Total 2
	b2/b3 deletion	b2/b3 duplication	b2/b4 duplication	gr/gr del. + b2/b4 duplication	gr/gr deletion	gr/gr duplication		
**E1b1b-M215**	6* (5.5)	3 (2.7)	-	-	-	1 (0.9)	99 (90.8)	109 (100.0)
**E1b1b1c1-M34**	-	-	-	-	1 (25.0)	-	3 (75.00)	4 (100.0)
**G2a-P15**	-	2 (16.7)	1 (8.3)	-	1 (8.3)	-	8 (66.67)	12 (100.0)
**H-M69**	-	-	-	-	-	-	9 (100.00)	9 (100.0)
**I1-M253**	-	-	1 (5.9)	-	1 (5.9)	1 (5.9)	14 (82.3)	17 (100.0)
**I2a (xI2a1)-P37.2**	-	-	1 (1.1)	-	5 (5.5)	-	85 (93.4)	91 (100.00)
**I2a1-M26**	-	-	-	-	-	-	2 (100.0)	2 (100.0)
**I2b1-M223**	-	-	1 (11.1)	-	1 (11.1)	-	7 (77.8)	9 (100.0)
**J1-M267**	-	-	-	-	-	-	7 (100.0)	7 (100.0)
**J2-M172**	-	4 (4.6)	1 (1.1)	1 (1.1)	-	1 (1.1)	80 (91.9)	87 (100.0)
**L-M20**	-	-	-	-	-	-	1 (100.0)	1 (100.0)
**N-M231**	1 (50.0)	-	-	-	-	-	1 (50.0)	2 (100.0)
**Q-M242**	-	-	-	-	-	-	2 (100.0)	2 (100.0)
**R1a-L62**	-	-	20^†^ (32.3)	-	2 (3.2)	6* (9.7)	34 (54.8)	62 (100.0)
**R1b-M343**	-	-	-	-	-	-	63 (100.0)	63 (100.0)
**R2-M124**	-	-	-	-	-	-	3 (100.0)	3 (100.0)
**T-M70**	-	-	2 (33.3)	-	2 (33.3)	-	2 (33.3)	6 (100.0)
**Total 1**	7 (1.4)	9 (1.8)	27 (5.6)	1 (0.2)	13 (2.7)	9 (1.8)	420 (86.4)	486 (100.0)

*P ≤ 0.01.

†*P* ≤ 0.001. The statistical analysis was carried out with Fischer exact test (row-wise comparisons) followed with Benjamin & Hochberg (BH) correction. The number of corrections with BH method was 25 (number of major haplogroups [n = 5] × number of rearrangements [n = 5] taken into account in the comparison).

We compared the microrearrangement frequencies of five major haplogroups to those of all other haplogroups. For achieving statistical power above 80%, the minimum sample size needed to observe at least medium effect size (proportion size) of a given microrearrangement for each of the five most frequent haplogroups should be 63, which was the case in our analysis. The statistical analysis was not performed for the remaining 12 haplogroups because of the small number of participants, even though some haplogroups were characterized by high frequencies of certain microrearrangements (Supplementary Figure 1(supplementary Figure 1) and Supplementary Figure 2(supplementary Figure 2)). The b2/b4 and gr/gr duplications were significantly associated with haplogroup R1a (*P* < 0.001 after BH correction; odds ratio [OR] 0.03525; 95% confidence interval [CI] 0.014-0.088; Power = 1 and *P* = 0.003 after BH correction; OR 0.06651; 95% CI 0.016-0.273; Power = 0.996, respectively), while b2/b3 was significantly associated with haplogroup E (*P* = 0.005 after BH correction; OR 0.04566; 95% CI 0.005-0.383). The gr/gr was most frequently detected in haplogroup I, showing a borderline statistical significance (*P* = 0.020), which was lost after BH correction for multiple testing (*P* = 0.101).

### Deletion subtypes defined according to the missing copies of DAZ and CDY1 genes

**Figure 1:**
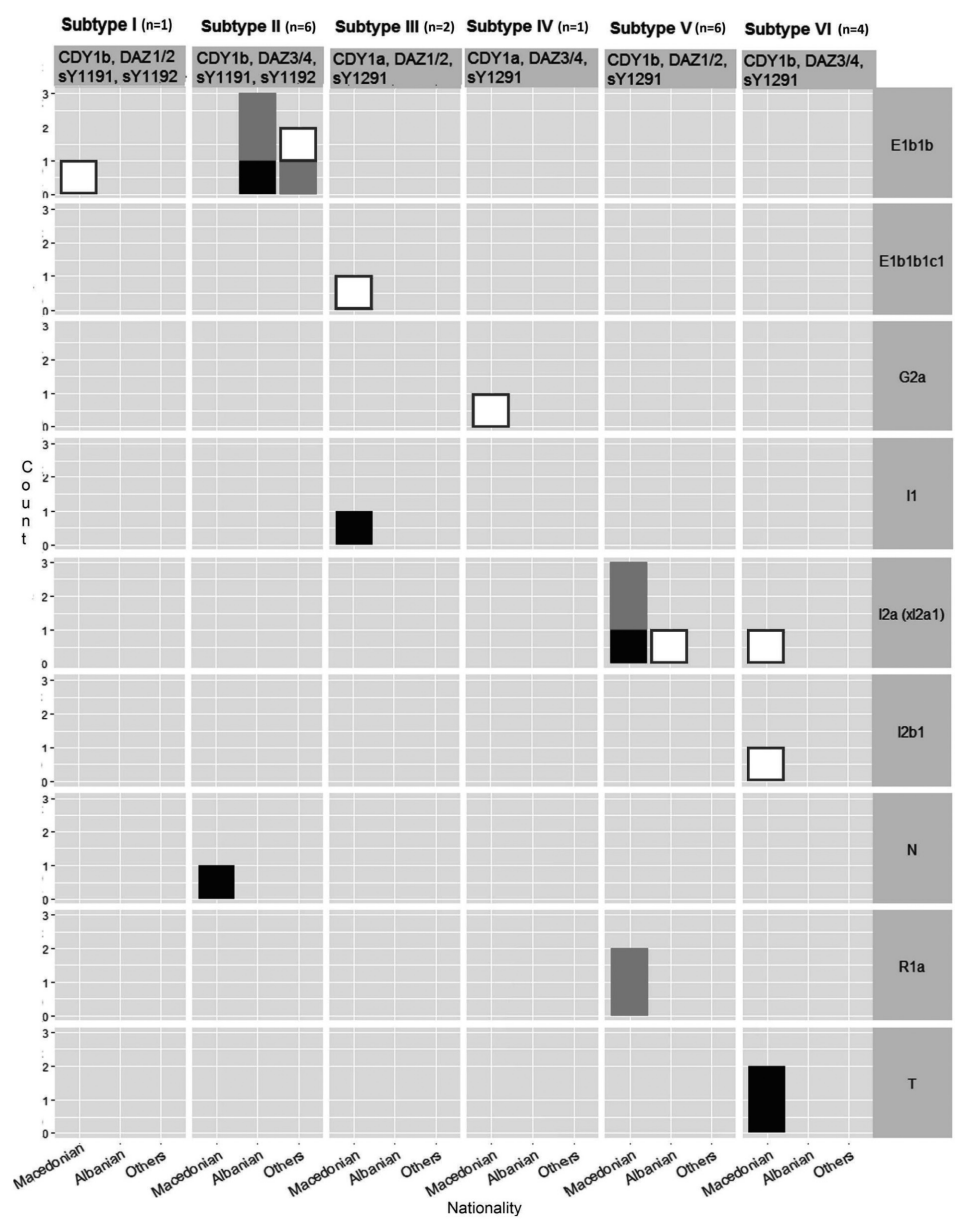
Four-variable graph showing the distribution of Y haplogroups, deletion subtypes, and ethnic background in azoospermic, oligozoospermic, and normozoospermic men. White – azoospermia (n = 154); gray – oligozoospermia (n = 184); black – normozoospermia (n = 148).

Among 20 participants with AZFc microdeletions we detected six different deletion patterns or subtypes: subtype I (CDY1b, DAZ 1/2, sY1191 and sY1192 deleted; n = 1), subtype II (CDY1b, DAZ 3/4, sY1191 and sY1192 deleted; n = 6), subtype III (CDY1a, DAZ 1/2 and sY1291 deleted; n = 2), subtype IV (CDY1a, DAZ 3/4 and sY1291 deleted; n = 1), subtype V (CDY1b, DAZ 1/2 and sY1291 deleted; n = 6), and subtype VI (CDY1b, DAZ 3/4 and sY1291 deleted, n = 4). The subtypes I and II were identified as b2/b3 deletions, while the other four subtypes were identified as gr/gr deletions. No significant differences were observed between the azoospermic/oligozoospermic and normozoospermic men (Figure 1).

Subtypes II and V were detected in not more than two different haplogroups in participants with different degrees of semen quality impairment. Subtypes III and VI were detected only among Macedonians with either azoospermia or normozoospermia, however they appeared in different haplogroups in participants with all degrees of semen quality impairment. Subtypes I and IV were detected only among azoospermic Macedonians in haplogroups G2 and E1b1b.

## DISCUSSION

Our results did not show a significant association between the degree of semen impairment and a specific microdeletion/microduplication, however we observed a significant association of b2/b4 and gr/gr duplications with haplogroup R1a and b2/b3 deletions with haplogroup E, implying that the Y chromosome background may confer susceptibility to AZFc microrearrangements.

A significant association between different types of AZFc rearrangements and impaired spermatogenesis, especially for gr/gr deletions, was reported in a number of studies, while others have failed to confirm such association (4,13,18,29,44-51). Several meta-analyses showed that gr/gr deletions carried an increased risk of severe spermatogenic failure (8,17,18). We observed similar frequencies of gr/gr deletions in both cases and controls, so we could find no evidence for the medium or large-size effect of the gr/gr deletions on the semen quality. Similar conclusions were made with respect to the b2/b3 deletions, and these results are in accordance with previously published studies for Italian and Moroccan population (49,52).

Regarding the AZFc duplications, studies in Asian populations reported an association between increased AZFc gene dosage and infertility (7,53,54), however studies conducted in Europe did not find such an association (50,52). Our results are in accordance with the results published for European populations, thus showing a high frequency of normozoospermic b2/b4 duplication carriers.

Since the two major ethnicities in North Macedonia genetically rarely admix, we included the ethnic origin and the haplogroups as elements that increase the resolution of the participants' classification. Our results showed a significant association of specific rearrangements with distinct haplogroups and with ethnic background within the haplogroups. Previous studies also found an increased prevalence of AZFc rearrangements within a particular haplogroup (8,55). Specifically, in our study b2/b3 deletions showed a significant association with haplogroup E, indicating that the genomic structure of this haplogroup might predispose the occurrence of the sole deletion. Haplogroup R1a exhibited the highest occurrence rate of b2/b4 duplications (32.26%), once again implying that the Y chromosome background influences the propensity for the occurrence of specific rearrangements. Furthermore, there was a significant difference in the frequency of Macedonian and Albanian HgR1a b2/b4 duplication carriers, suggesting that the R1a Albanians are more prone to this type of rearrangement. It is important to point out that our sample size prevented us from assessing the association of less prevalent Y haplogroups with partial deletions/duplications.

The limitations of the study include retrospective design, a highly selected patient population, and the fact that the study was based on a single center experience. Because of the small effect size, our statistical analyses were underpowered when assessing the association of microrearrangements with impaired semen quality. This warrants the cooperation of different centers to increase the number of participants. Another limitation is that we could not examine the father/son pairs, which prevented us from determining whether the occurrence of the rearrangements in a distinct haplogroup emerged *de novo* or was inherited. If we had been able to investigate the Y chromosome of our patients’ fathers, we would have been capable to directly determine the influence of the partial deletions/duplications on the fertility fitness. However, the high occurrence rate of some rearrangements within particular haplogroups, for example the gr/gr and b2/b4 duplications in the haplogroup R1a, indicate that they are probably naturally transmitted and have neutral or even favorable influence on the semen quality.

Further on, we made efforts to resolve the AZFc deletion patterns to the most attainable resolution using a combination of different STS and gene/probe dosage markers. After subtype classification using haplogroup, ethnic, and spermatogenic status, we noticed that subtypes CDY1b, DAZ 3/4, sY1191, sY1192 and CDY1b, DAZ 1/2 and sY1291 were characteristic for distinctive haplogroups and were not present in more than two haplogroups. Additionally, subtypes CDY1b, DAZ 1/2, sY1191, sY1192 and CDY1a, DAZ 3/4, and sY1291 emerging in haplogroups E1b1b and G2 were present only in participants with azoospermia. These findings suggest that the increase in the resolution of subtype and haplogroup analysis for the AZFc partial deletions could identify the damaging subtypes. The referent, detailed structure of the Y chromosome is known only for the R1 haplogroup, while the complete sequence and structural organization of other Y chromosomes is currently unknown. Potentially harmful microdeletions could be detected by comparing the detailed structural organization of the Y chromosomes carrying gr/gr or b2/b3 deletions in haplogroups known to be inherent deletion carriers (haplogroups N and D2b) with Y chromosomes carrying the same deletions in haplogroups associated with impaired semen quality. The currently available technologies, such as short read massive parallel sequencing, are unsuitable and ineffective in sequencing the large palindromic sequences present in the AZF regions of the Y chromosomes, especially the AZFc region. The emergence of third-generation sequencing techniques, involving reads that can be tens of kilobases long, could overcome the technical difficulties and provide access to more repeated regions of the MSY (56).
