# Supplementary material for: Y-chromosome haplogroup architecture confers susceptibility to azoospermia factor c microrearrangements: a retrospective study
Source: Croat Med J. 2019 Jun;60(3):273–83. doi: 10.3325/cmj.2019.60.273 (PMC6563173; doi:10.3325/cmj.2019.60.273)
Supplement: Supplementary Figure 1 [file CroatMedJ_60_s001.pdf]

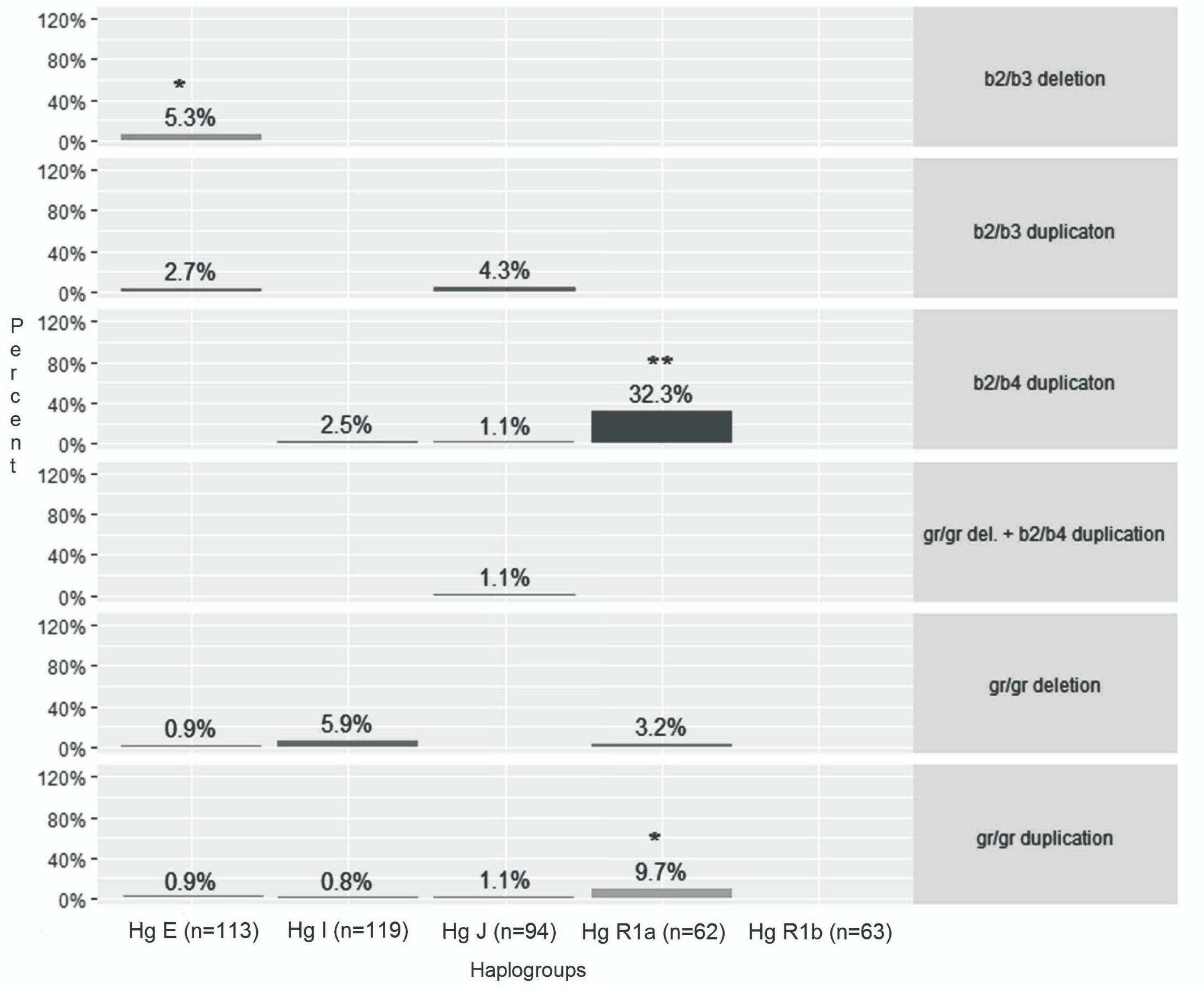

Supplementary Figure 1. Distribution of the AZFc rearrangements among the five most common Y haplogroups. The statistically significant results were obtained by comparison of the frequencies of the detected rearrangements of the five major Y lineages to the deletion/duplication frequencies of all other haplogroups. The asterisk above the bars designates statistically significant results. *p ≤0.01; ** p ≤0.001
