# Supplementary material for: Y-chromosome haplogroup architecture confers susceptibility to azoospermia factor c microrearrangements: a retrospective study
Source: Croat Med J. 2019 Jun;60(3):273–83. doi: 10.3325/cmj.2019.60.273 (PMC6563173; doi:10.3325/cmj.2019.60.273)
Supplement: Supplementary Figure 2 [file CroatMedJ_60_s002.pdf]

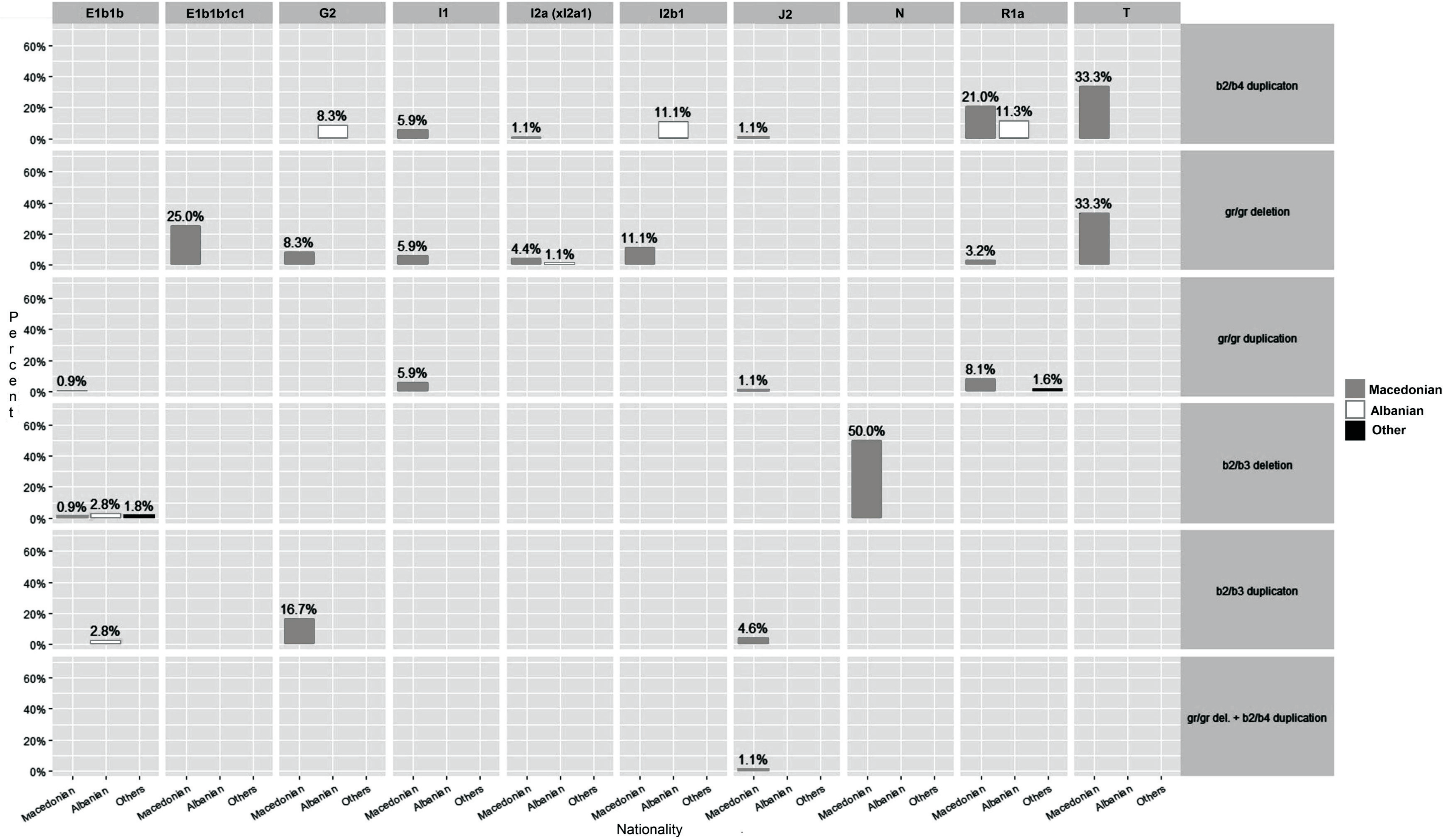

Supplementary Figure 2. Detailed three variable figure, showing the distribution of deletions/duplications within the detected haplogroups and ethnicities.
